# Enhancing Magnetic Micro- and Nanoparticle Separation with a Cost-Effective Microfluidic Device Fabricated by Laser Ablation of PMMA

**DOI:** 10.3390/mi15081057

**Published:** 2024-08-22

**Authors:** Cristian F. Rodríguez, Paula Guzmán-Sastoque, Carolina Muñoz-Camargo, Luis H. Reyes, Johann F. Osma, Juan C. Cruz

**Affiliations:** 1 Department of Biomedical Engineering, Universidad de los Andes, Cra. 1E No. 19a-40, Bogotá 111711, Colombia; cf.rodriguez@uniandes.edu.co (C.F.R.); pa.guzmans@uniandes.edu.co (P.G.-S.); c.munoz2016@uniandes.edu.co (C.M.-C.); jf.osma43@uniandes.edu.co (J.F.O.); 2 Neuroscience Group of Antioquia, Cellular and Molecular Neurobiology Area, School of Medicine, University of Antioquia, Medellin 050010, Colombia; 3 Grupo de Diseño de Productos y Procesos (GDPP), Department of Chemical Engineering, Universidad de los Andes, Cra. 1E No. 19a-40, Bogotá 111711, Colombia; lh.reyes@uniandes.edu.co; 4 Department of Electrical and Electronic Engineering, Universidad de los Andes, Cra. 1E No. 19a-40, Bogotá 111711, Colombia

**Keywords:** magnetite, microfluidic, separation, purification, CFD, SWOT

## Abstract

Superparamagnetic iron oxide micro- and nanoparticles have significant applications in biomedical and chemical engineering. This study presents the development and evaluation of a novel low-cost microfluidic device for the purification and hyperconcentration of these magnetic particles. The device, fabricated using laser ablation of polymethyl methacrylate (PMMA), leverages precise control over fluid dynamics to efficiently separate magnetic particles from non-magnetic ones. We assessed the device’s performance through Multiphysics simulations and empirical tests, focusing on the separation of magnetite nanoparticles from blue carbon dots and magnetite microparticles from polystyrene microparticles at various total flow rates (TFRs). For nanoparticle separation, the device achieved a recall of up to 93.3 ± 4% and a precision of 95.9 ± 1.2% at an optimal TFR of 2 mL/h, significantly outperforming previous models, which only achieved a 50% recall. Microparticle separation demonstrated an accuracy of 98.1 ± 1% at a TFR of 2 mL/h in both simulations and experimental conditions. The Lagrangian model effectively captured the dynamics of magnetite microparticle separation from polystyrene microparticles, with close agreement between simulated and experimental results. Our findings underscore the device’s robust capability in distinguishing between magnetic and non-magnetic particles at both micro- and nanoscales. This study highlights the potential of low-cost, non-cleanroom manufacturing techniques to produce high-performance microfluidic devices, thereby expanding their accessibility and applicability in various industrial and research settings. The integration of a continuous magnet, as opposed to segmented magnets in previous designs, was identified as a key factor in enhancing magnetic separation efficiency.

## 1. Introduction

Micro- and nanoparticles have become integral components across various industries, particularly in chemical and biomedical engineering [1,2,3,4,5,6,7,8]. This widespread integration is driven by the unique and advantageous properties that materials exhibit at the micro- and nanoscales. In the biomedical field, nanoparticles are of particular interest due to their promising applications in drug delivery and disease diagnostics, presenting a frontier for innovation in medical treatments and technologies [9,10,11,12]. Nanomaterials employed for drug delivery can be broadly categorized into organic, hybrid, and inorganic types [13,14,15,16]. Organic nanoparticles include liposomes, solid lipid nanoparticles, polymeric nanoparticles, and polymeric micelles [17,18,19,20]. Hybrid nanoparticles comprise those with both organic and inorganic components, such as Metal-Organic Frameworks (MOFs) [21,22]. Inorganic nanoparticles encompass a range of materials including gold nanoparticles, quantum dots, silver nanoparticles, and iron oxide nanoparticles [23,24,25,26,27]. Of particular interest is magnetite, an iron oxide nanoparticle with superparamagnetic properties [28,29,30]. Superparamagnetic nanoparticles are valuable in applications such as sample hyperconcentration for compound detection and disease diagnosis [31,32,33]. The unique magnetic properties of these particles allow for their manipulation using external magnetic fields, enabling precise control and targeting in various applications.

Significant advancements have been made in the techniques for magnetic separation and hyperconcentration of micro- and nanoparticles [34,35,36]. Notably, microfluidic approaches have emerged as increasingly vital in the domains of particle purification, washing, and hyperconcentration [37,38]. This prominence is attributed to the unique advantages of microfluidic systems: they provide precise control over fluid dynamics at the microscale, facilitate high-accuracy particle manipulation and sorting, and enable the integration of multiple functional processes within a single microfluidic chip [39,40].

Microfluidic devices can be fabricated from a variety of materials, including polydimethylsiloxane (PDMS), silicon, glass, and thermoplastics such as polystyrene (PS), polycarbonate (PC), cyclic olefin copolymers (COC). Various manufacturing techniques, such as soft lithography, 3D printing, and laser ablation, are employed in their production [41,42,43,44,45].

Recently, numerous microfluidic devices have emerged with the capability to purify, concentrate, and separate samples using magnetic fields. The separation and purification of magnetic nanoparticles are of great interest in many industries, including the analysis of chemical agents in contaminated waters. For example, Ortegón et al. utilized a novel microextraction coupled with furnace atomic absorption spectroscopy [33]. Their study demonstrated the feasibility of improving the adsorption process on a magnetic sorbent material, resulting in a significant preconcentration factor and enhanced sensitivity for arsenic detection.

In diagnostic applications, the use of magnetic microfluidic devices for hyperconcentration and separation has also shown promising results. Soroush et al., for instance, presented a novel magneto-plasmonic biosensor for the immunodetection of antigens in minute sample volumes [46]. Their system combined spherical gold nanoparticles and magnetic particles, both conjugated to goat anti-rabbit IgG antibodies, to recognize a model target, rabbit IgG. The magnetic particles served as the capture probe, allowing the sandwich immunocomplex to form with the target analyte and detection probe to be separated from unbound Au nanoparticle conjugates by applying an external magnetic field. 

Although numerous microfluid devices have emerged for various applications, a significant number of these devices are manufactured using cleanroom techniques, posing a substantial limitation for laboratories lacking cleanroom facilities [47].

In light of these developments and challenges, this study aims to develop a novel microfluidic device specifically designed for the purification and hyperconcentration of magnetic micro- and nanoparticles. Our approach combines computational modeling, low-cost fabrication techniques, and rigorous experimental validation to create a high-performance device that is accessible to a wider range of researchers and applications. To achieve this, we first conducted in silico simulations using COMSOL Multiphysics to optimize the magnetic purification process. Following the optimization, the device was fabricated using a cost-effective laser ablation technique in PMMA. This fabrication method represents a significant departure from traditional cleanroom-based approaches, potentially broadening the accessibility of such devices.

The performance of the microfluidic device was assessed through its ability to separate magnetite nanoparticles from blue carbon dots and magnetite microparticles from polystyrene microparticles. Both computational simulations and empirical tests were conducted at various total flow rates (TFRs) to comprehensively characterize the device’s performance under different operating conditions.

## 2. Materials and Methods

### 2.1. Materials

Table 1 presents the materials employed. All chemicals were used as received without further purification.

### 2.2. Computational Modeling of Micro- and Nanoparticle Separation Dynamics 

The magnetic separation process was modeled using the Euler-Lagrange approach in the software COMSOL Multiphysics 6.2 (COMSOL Inc., Stockholm, Sweden). This approach, governed by Newton’s second law of motion (Equation (1)), treats particles as individual entities.

(1) $$Ft=\frac{d\left(m_{p}*v\right)}{dt}$$

where $m_{p}$ is the particle mass, v is the velocity, and Ft is the sum of all forces acting on the particles. Thus, the particles experience the effects of both the magnetic field and the fluid flow forces within the microfluidic devices. To model the effect of the fluid on the nanoparticles, the fluid was treated as a laminar flow governed by the Navier–Stokes equation (Equation (2)) and the continuity equation (Equation (3)), representing momentum conservation and mass conservation, respectively.

(2) $$\rho \left(u\cdot \nabla \right)u=\nabla \cdot \left[-pI+\mu \left(\nabla u+{\left(\nabla u\right)}^{T}\right)\right]+F$$

(3) ρ∇⋅u=0

where *u* is the fluid velocity, I is the identity matrix, p is the fluid pressure, and ρ is the fluid density. The effect of the fluid on the particles was modeled by including the drag force, as represented by Equation (4):

(4) $$Fd=\left(u-v\right)*\frac{m_{p}}{\tau_{p}}$$

where Fd is the drag force, v is the particle velocity, $m_{p}$ is the particle mass, $\tau_{p}$ is the Lagrangian time scale, and *u* is the velocity field. The effect of the magnetophoretic force was considered using Equation (5). This equation is critical in quantifying the influence of a magnetic field on the movement of magnetic particles.

(5) $$F_{m}=2\pi r_{p}^{3}\mu_{0}\mu_{r}\left(\frac{\mu_{r,p}-\mu_{\Gamma }}{\mu_{r}+2\mu_{r}}\right)\nabla H^{2}$$

where $r_{p}$ is the particle radius, $\mu_{0}$ is the permeability of free space, $\mu_{r}$ is the fluid’s relative permeability, $\mu_{r,p}$ is the particle’s relative permeability, and H is the magnetic field strength. The magnetic field was determined by solving Maxwell’s equations (Equations (6) and (7)):

(6) $$H=-\nabla V_{m}$$

**Figure 1:**
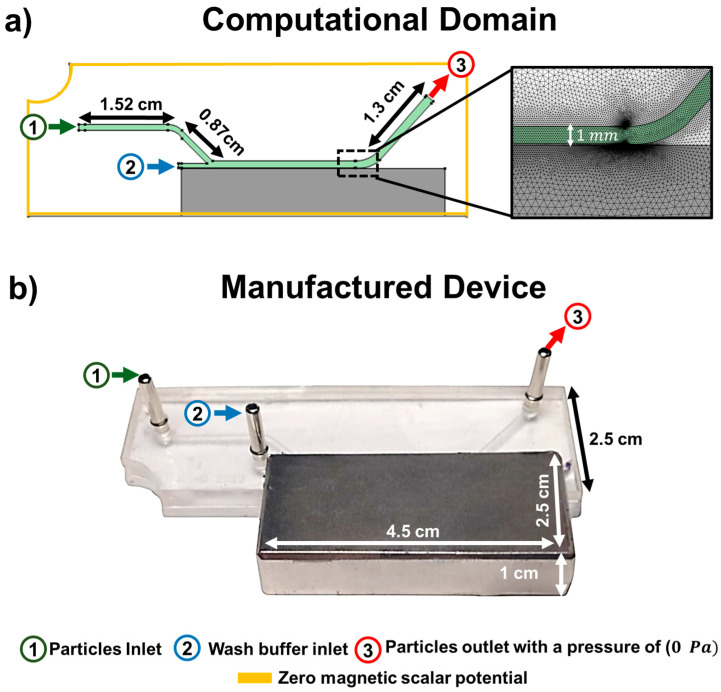
Computational domain and boundary conditions. (**a**) Computational domain, and mesh used for the simulation. (**b**) Microfluidic device manufactured in PMMA using laser ablation. The particle inlet is shown in green and labeled ‘1’, and the water buffer inlet, facilitating the washing and separation of magnetic from non-magnetic particles, is in blue and labeled ‘2’. The outlet, marked in red and denoted by ‘3’, is where non-magnetic particles exit first, followed by magnetic particles after the magnet is removed.

(7) ∇⋅B=0

where $V_{m}$ is the magnetic scalar potential, and B the magnetic flux density determined by Equation (8):

(8) $$B=\mu_{0}\mu_{r}H+B_{r}$$

where $\mu_{0}$ is the permeability of free space, $\mu_{r}$ is the fluid’s relative permeability, and $B_{r}$ is the remanent flux density. To solve the system of equations, the domain was discretized into 53,520 tetrahedral elements and 1870 boundary elements. This level of meshing ensured the convergence of the solution. The mesh configuration is depicted in Figure 1.

### 2.3. Solvers and Computational Simulation

The Multiphysics simulation of the microfluid device was carried out using a sequential approach. Initially, the magnetic field and the fluid flow within the microchannels were computed using a stationary study. This part of the simulation employed the Multifrontal Massively Parallel Sparse Direct Solver (MUMPS), which effectively handles large sparse systems of linear equations.

Upon obtaining the stationary solutions for both the magnetic field and fluid flow, these results were then integrated into the particle tracing model. This coupling process was executed using the Generalized Minimum Residual (GMRES) solver in a time-dependent framework, allowing for the dynamic interaction between the magnetic and fluid fields with the particle trajectories.

The parameters utilized for these simulations are detailed in Supplementary Table S1. This includes the mean and standard deviation for the particle diameter, which were characterized by a normal distribution probability density function as described by Equation (9):

(9) $$f\left(d_{p}\right)=\frac{1}{\sigma \sqrt{2\pi }}{\mathrm{e}}^{-\frac{1}{2}{\left(\frac{d_{p}-\mu }{\sigma }\right)}^{2}}$$

where *μ* denotes the mean diameter and *σ* signifies the standard deviation of the particle diameters. Particles were introduced into the microsystem as a boundary condition at inlet 1. Meanwhile, a washing buffer was introduced at inlet 2 to enhance the separation between particles. The particles and the washing buffer were then directed towards the outlet. These boundary conditions, which encompass the zero magnetic scalar potential for solving the magnetic field, are depicted in Figure 1.

### 2.4. Manufacture

**Figure 2:**
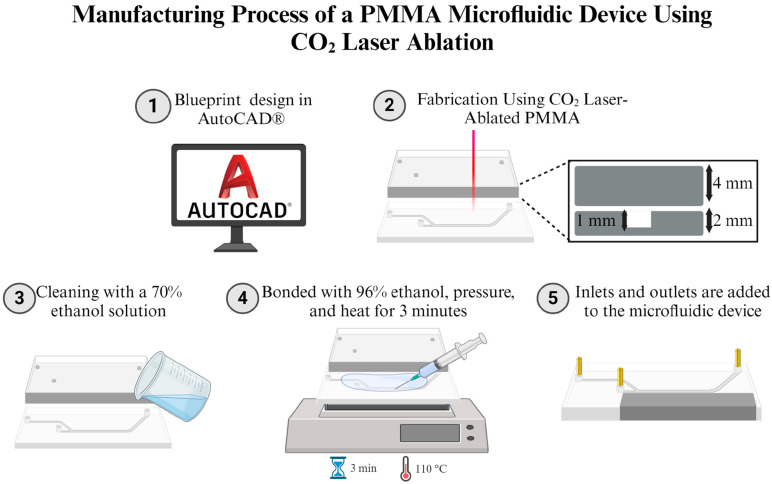
Manufacturing process of microfluidic devices using CO_2_ laser ablation in PMMA. (1) The microfluidic device is manufactured in AutoCAD (AutoDesk Inc., Mill Valley, CA, USA). (2) The design is transferred to a CO_2_ laser system for engraving and cutting. A 2 mm-thick PMMA sheet is engraved to a depth of 1 mm to create the microfluidic channels, while a 4 mm-thick PMMA sheet is cut to form the inlets and outlets. (3) Then, PMMA layers are cleaned with a 70% ethanol solution to remove any residues. (4) Next, layers are bonded using 96% ethanol, pressure, and heat at 110 °C for 3 min. (5) Finally, the inlets and outlets are assembled into the microfluidic device.

The microfluidic device was manufactured using a low-cost technique involving laser ablation of polymethyl methacrylate (PMMA). The materials and their costs are described in Table 2. The process, illustrated in Figure 2, began with the creation of the microfluidic device design using AutoCAD software 2022.1.5 (AutoDesk Inc., Mill Valley, CA, USA). In the design, areas to be engraved were marked in black, while cutting lines were denoted in red to differentiate between ablation and cutting processes. The design was then transferred to PMMA sheets using a TROTEC^®^ laser cutting system (Marchtrenk, Austria). For the cutting process, the laser power was set to 120 W with a speed of 0.010 m/s, while for engraving, the power was reduced to 18 W with an increased speed of 0.355 m/s. These parameters were carefully optimized to achieve precise channel dimensions and a smooth surface finish.

Following the laser processing, the PMMA sheets were thoroughly cleaned with a 70% ethanolic solution to remove any surface residues and debris from the ablation process. This cleaning step is crucial to ensuring clean bonding surfaces and preventing contamination in the final device. The cleaned PMMA sheets were then bonded using a thin application of 96% ethanol. To create a strong, leak-free seal between the PMMA layers, the sheets were placed under constant pressure and heated to 110 °C for 3 min in a thermal bonding process. After the heating process, the microfluidic devices were removed from the hot plate and allowed to cool under constant pressure. This controlled cooling helps prevent warping and ensures the dimensional stability of the device. Once the microfluidic system reached ambient temperature, the inlets were carefully integrated, involving the attachment of tubing or connectors to the inlet and outlet ports to facilitate fluid introduction and collection.

### 2.5. Synthesis of Micro- and Nanoparticles of Magnetite

**Figure 3:**
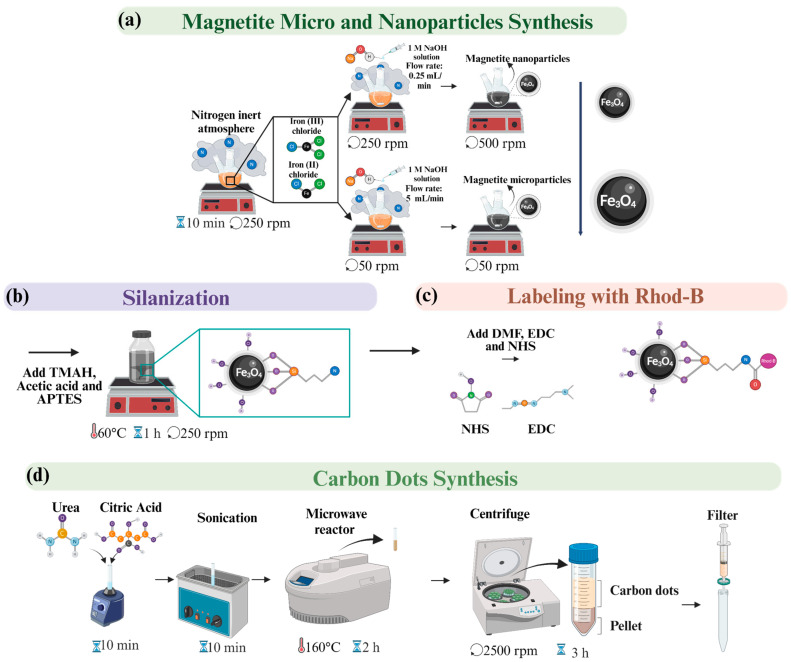
Synthesis and functionalization of magnetite micro- and nanoparticles. (**a**) Schematic of the magnetite micro- and nanoparticle synthesis using the coprecipitation technique. The process involves the preparation of an iron chloride solution, followed by the addition of NaOH. Rapid addition yields micro-sized particles (~2405 nm), while slow, dropwise addition results in nano-sized particles (~155 nm). (**b**) Silanization of magnetite nanoparticles to functionalize their surface and facilitate further modifications. (**c**) Subsequent labeling of silanized magnetite nanoparticles with rhodamine B (Rhod-B). (**d**) Synthesis of carbon dots through a separate process involving heating, sonication, and filtration, yielding purified carbon dots.

The synthesis of both micro- and nanoparticles of magnetite was achieved using a modified coprecipitation technique as described by Guzmán et al. [48] involving precise control over the reactant solutions and reaction conditions (Figure 3a). Initially, an iron chloride solution was prepared by dissolving 1.988 g of ferrous chloride (FeCl_2_) and 5.406 g of ferric chloride (FeCl_3_) in 100 mL of deionized water at 4 °C, aiming for a Fe^2+^: Fe^3+^ molar ratio of 1:2 (0.01 to 0.02 moles, respectively). This solution was thoroughly mixed at 300 RPM until homogeneous and then sonicated for 5 min to ensure complete dissolution of all solids.

In parallel, a sodium hydroxide (NaOH) solution was prepared by dissolving 3.2 g of NaOH in 100 mL of Type I water to create a 0.08 molar solution. Both solutions were cooled to 2 °C before mixing to better control the reaction kinetics. The chloride solution was then deoxygenated by bubbling nitrogen through it at a flow rate of 0.5 L/min for 10 min while stirring at 100 RPM. This step was critical to creating an oxygen-free environment, preventing unwanted oxidation reactions during nanoparticle formation.

The synthesis route then diverged based on the desired particle size. For micro-sized particles, the NaOH solution was added quickly to the chloride mixture, inducing rapid precipitation. Conversely, for nano-sized particles, the NaOH solution was added dropwise into the vigorously stirred chloride mixture at 250 RPM, and the reaction was allowed to proceed under these conditions for 30 min. This slower addition and sustained agitation helped in achieving a finer particle size.

Finally, both micro- and nano-sized particles were subjected to a stringent purification process involving five wash cycles. The initial three cycles used a 1.5% *w/v* NaCl solution, followed by two additional washes with Type I water. Each washing step included sonication for five minutes to disperse any agglomerates and remove surface impurities effectively, ensuring the purity and stability of the final magnetic particle products.

The circular shape of the magnetic nanoparticles was verified through Transmission Electron Microscopy (TEM) (Figure S1A). The hydrodynamic sizes of the magnetite nano- and microparticles were determined using dynamic light scattering (DLS) with a Zeta-Sizer Nano ZS instrument (Malvern, UK), resulting in sizes of 155 nm (Figure S1B) for the nanoparticles and 2405 nm (Figure S1C) for the microparticles.

#### 2.5.1. Silanization and Labeling of the Magnetic Nanoparticles (MNPs)

To effectively characterize the microfluidic magnetic separation system, we modified the magnetic nanoparticles (MNPs) to emit red-light fluorescence. This modification involved a two-step process: silanization of the MNPs followed by labeling with rhodamine B (Figure 3b,c).

##### Silanization Process

Initially, 100 mg of MNPs were suspended in 40 mL of Type I water and sonicated for 5 min to ensure uniform dispersion. Following sonication, 250 µL of tetramethylammonium hydroxide (TMAH) was added dropwise to the suspension. The mixture was then sonicated for an additional minute and stirred at 100 RPM for 3 min to promote surface modification. Then, 50 µL of glacial acetic acid was introduced into the mixture, followed by further sonication for one minute and stirring for 3 more minutes. Subsequently, 1 mL of a 20% (*v*/*v*) solution of (3-Aminopropyl) triethoxysilane (APTES) was added dropwise while maintaining the temperature at 60 °C and stirring at 100 RPM. The reaction was allowed to proceed for one hour at 220 RPM to ensure a thorough coating of the nanoparticle surface with APTES.

##### Rhodamine B Labeling

For the labeling, an amino-carboxyl functionalization technique was utilized. Specifically, 6.41 × 10^−5^ mol of rhodamine B, 12.3 mg of EDC, and 7.4 mg of NHS were dissolved in 2 mL of dimethylformamide (DMF) and then diluted in 3 mL of Type I water. This mixture was heated to 37 °C under continuous magnetic stirring for 15 min to activate the carboxyl group of rhodamine B. The pre-activated solution was then combined with 40 mL of the magnetite nanoconjugates solution (2.5 316 mg/mL), sonicated for 5 min, and left under constant mechanical stirring at 200 RPM for 24 h to achieve thorough conjugation.

##### Purification Process

Both post-silanization and post-labeling, the particles underwent a rigorous purification protocol involving five wash cycles to remove unreacted chemicals and by-products. The first three cycles utilized a 1.5% *w*/*v* NaCl solution, which helped to remove loosely bound APTES molecules and stabilize the silanized surface. The final two washes were performed with Type I water to remove any residual salts. Each washing step incorporated sonication for five minutes, which served to effectively disperse any agglomerates that may have formed during the reaction and remove surface impurities.

The purification process was not only crucial after silanization but was also repeated following the subsequent labeling step. This ensured the removal of any unbound rhodamine B molecules, leaving only the fluorophores covalently attached to the silanized nanoparticle surface. The identical washing protocol was employed post-labeling, maintaining consistency in the purification process and ensuring the purity and stability of the final fluorescently labeled magnetic nanoparticles.

This meticulous process of silanization, labeling, and purification resulted in magnetic nanoparticles with a stable, fluorescent surface coating. These modified nanoparticles were crucial for the subsequent evaluation of the microfluidic magnetic separation system, allowing for clear visualization and quantification of the separation process.

#### 2.5.2. Synthesis of Blue Carbon Dots

The synthesis of blue carbon dots (Figure 3d) was carried out using a method adapted from Miao et al. [49]. The initial step involved dissolving 0.3 mmol of citric acid and 3 mmol of urea in 3 mL of dimethylformamide (DMF). To ensure complete mixing of the reactants, the solution was stirred at 200 rpm until homogeneous and then sonicated for 10 min. The thoroughly mixed solution was then transferred to a microwave reactor (Monowave 50, Anton Paar, Graz), where it was rapidly heated to 180 °C and maintained at this temperature for 2 h to promote the reaction and formation of carbon dots. Following the microwave-assisted synthesis, the reaction mixture was subjected to centrifugation at 2500 rpm for 2 h to effectively separate solid residues from the supernatant containing the carbon dots. To further purify the solution and remove any residual clusters, the supernatant was filtered through 0.22 µm syringe filters. This filtration was crucial for obtaining a clear solution of purified blue carbon dots, making them suitable for subsequent use in the experimental separation tests.

### 2.6. Experimental Separation Tests

#### 2.6.1. Nanoparticle Separation

The evaluation of the magnetic separator device’s efficacy in purifying magnetic nanoparticles involved a series of experimental separation tests designed to assess the device under various operational conditions. A mixture comprising magnetic nanoparticles labeled with rhodamine B and blue carbon dots, both at a concentration of 0.1 mg/mL, was prepared to facilitate this evaluation. This composite was introduced into the magnetic microfluidic device to analyze its separation efficiency.

Three different total flow rates (TFRs) of 2, 20, and 200 mL/h were tested, maintaining a 1:1 flow rate ratio between the particle inlet and the wash buffer inlet, which used Type II water. The aim was to purify the suspension, and the wash buffer was pumped using a KDS-100 syringe infusion pump from W.P. Instruments (Holliston, MA, USA). Following the separation process, 1 mL of purified particle suspension was collected for analysis. The particle inlet was then replaced with an additional wash buffer inlet to eliminate any residual particles, with the first 500 µL being discarded as waste.

After the initial collection, the magnet was detached from the microfluidic device, and any particles remaining within were collected for further analysis. The efficacy of the separation process was quantitatively assessed by analyzing the two collected vials using a spectrofluorometer (0239D-2219 FluoroMax Plus C, Horiba, Miyanohigashi, Japan). Measurements were taken at excitation wavelengths of 350 nm and 546 nm, corresponding to emission wavelengths of 425 nm and 570 nm, respectively.

For a robust analysis of the separation performance, key metrics including true positives, false positives, true negatives, and false negatives were calculated. These metrics provided a comprehensive evaluation of the device, framing the process as a binary separation challenge. In this setup, red-labeled magnetite particles were designated as the targets (positives), while the blue carbon dots were considered non-targets (negatives). To further assess the efficacy of the magnetic separator device in purifying magnetic nanoparticles, the performance metrics of recall, precision, and accuracy were evaluated. Recall, also known as sensitivity or true positive rate, was calculated using Equation (10):

(10) $$Recall=\frac{I_{RS\;570}-I_{B\;570}}{\left(I_{RS\;570}-I_{B\;570}\right)+(I_{\;WB\;570}-I_{B\;570})}$$

where $I_{RS\;570}$ represents the intensity of the fluorescence signal at 570 nm in the recollected sample, $I_{B\;570}$ is the intensity of the background (only water) at 570 nm, and $I_{\;WB\;570}$ is the intensity at 570 nm of the washing buffer. Precision, also known as positive predictive values, measures the proportion of correctly identified target particles (true positives) out of all particles identified as targets. Precision is calculated using Equation (11):

(11) $$Precision=\frac{(I_{RS\;570}-I_{B\;570)\;}}{\left(I_{RS\;570}-I_{B\;570}\right)+(I_{RS\;425}-I_{B\;425})}$$

where $I_{RS\;570}$ represents the intensity of the fluorescence signal at 570 nm in the recollected sample, $I_{B\;570}$ is the intensity of the background (only water) at 570 nm, $I_{B\;425}$ is the intensity of the background (only water) at 425 nm, and $I_{\;RS\;425}$ is the intensity at 425 nm of the recollected sample. Accuracy was calculated using Equation (12):

(12) $$Accuracy=\frac{\left(I_{RS\;570}-I_{B\;570})+(I_{\;WB\;425}-I_{B\;425}\right)}{\left(I_{RS\;570}-I_{B\;570})+(I_{\;WB\;425}-I_{B\;570})+(I_{\;RS\;425}-I_{B\;425})+(I_{\;WB\;570}-I_{B\;570}\right)}$$

where $I_{RS\;570}$ represents the intensity of the fluorescence signal at 570 nm in the recollected sample, $I_{\;RS\;425}$ is the intensity at 425 nm of the recollected sample, $I_{\;WB\;570}$ is the intensity at 570 nm of the washing buffer, $I_{\;WB\;425}$ is the intensity at 425 nm of the washing buffer, $I_{B\;570}$ is the intensity of the background (only water) at 570 nm, and $I_{B\;425}$ is the intensity of the background (only water) at 425 nm.

#### 2.6.2. Microparticle Separation

The capacity of the microfluidic device for separating microparticles was evaluated by assessing its ability to accurately distinguish magnetite microparticles from polystyrene microparticles labeled with rhodamine B (B MV-F02, Microvec, Piczów, Poland). During the evaluation test, a mixture of magnetite and polystyrene microparticles was introduced into the microfluidic device, both at a concentration of 0.1 mg/mL. The total flow rate was varied at 2, 20, 200 mL/h, utilizing the same KDS-100 syringe infusion pump from W.P. Instruments (Holliston, MA, USA) as in the nanoparticle separation tests.

For each separation test, a 1 mL sample of the separated microparticles was collected. To eliminate any residual particles after collecting the initial 1 mL sample, the particle inlet was replaced with a wash buffer inlet, and the first 500 μL of the wash buffer was discarded as waste. Subsequently, the magnet was detached from the microfluidic device to collect any remaining microparticles within the system.

The collected samples were then visualized using a fluorescence microscope (Axio, ZEISS, Jena, Germany) with a 10× objective lens, and the samples were excited at 530 nm. This imaging process allowed for a clear distinction between the non-fluorescent magnetite microparticles and the fluorescent polystyrene microparticles. The separation efficiency was analyzed as a binary separation problem, with magnetite microparticles defined as positives and polystyrene microparticles as negatives. It was evaluated using the parameters of recall, precision, and accuracy, counting the number of microparticles of magnetite, and the number of polystyrene microparticles. Recall was calculated using Equation (13):

(13) $$Recall=\frac{{MP}_{RS}}{\left({MP}_{RS}\right)+({MP}_{WB})}$$

where ${MP}_{RS}$ represents the number of magnetite particles in the recollected sample, and ${MP}_{WB}$ is the number of magnetite particles in the washing buffer. Precision, also known as positive predictive values, measures the proportion of correctly identified target particles (true positives) out of all particles identified as targets. Precision is calculated using Equation (14):

(14) $$Precision=\frac{{MP}_{RS}}{\left({MP}_{RS}\right)+({PP}_{RS})}$$

where ${MP}_{RS}$ represents the number of magnetite particles in the recollected sample, and ${PP}_{RS}$ is the number of polystyrene particles in the recollected sample. Accuracy was calculated using Equation (15):

(15) $$Accuracy=\frac{{MP}_{RS}+{PP}_{WB}}{{MP}_{RS}+{PP}_{WB}+{MP}_{WB}+{PP}_{RS}}$$

where ${MP}_{RS}$ represents the number of magnetite particles in the recollected sample, ${PP}_{RS}$ is the number of polystyrene particles in the recollected sample, ${MP}_{WB}$ is the number of magnetite particles in the washing buffer, and ${PP}_{WB}$ is the number of polystyrene particles in the washing buffer.

## 3. Results and Discussion

### 3.1. Nanoparticle Separation

To assess the device’s proficiency in nanoscale particle separation, we conducted a comprehensive study focusing on the differentiation of blue carbon dots from magnetite nanoparticles. This separation challenge was treated as a binary classification problem, with magnetite identified as the target (positive) and quantum dots as the non-target (negative) entities. The efficacy of the separation process was rigorously analyzed through both computational simulations and empirical experiments across a spectrum of total flow rates (TFRs) of 2, 20, and 200 mL/h.

**Figure 4:**
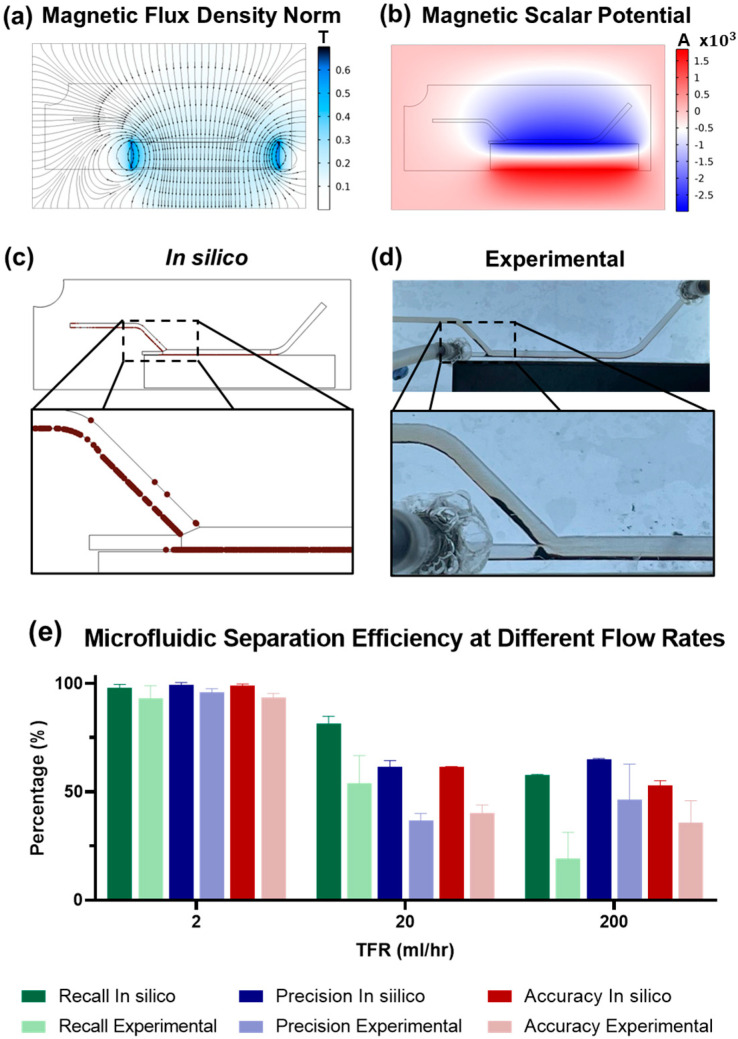
Evaluation of magnetic nanoparticle separation microfluidic device. (**a**) Simulated magnetic flux density distribution around the continuous magnet. (**b**) Distribution of the magnetic scalar potential across the microfluidic device, indicating areas of maximum potential (up to ±2 amperes). (**c**) Trajectory simulation of magnetic nanoparticles in the microfluidic channel, demonstrating their response to the applied magnetic field. (**d**) Photographic evidence of nanoparticle retention within the microfluidic device, aligning with areas of high magnetic flux density. (**e**) Comparative bar graph showcasing recall, precision, and accuracy metrics for nanoparticle separation at varying total flow rates (2, 20, and 200 mL/h), as obtained from both in silico simulations (dark shades) and experimental results (light shades).

The outcomes of these evaluations are encapsulated in Figure 4, which illustrates the simulated norm of the magnetic flux density, highlighting regions with peak intensities reaching 0.6 T. Concurrently, Figure 4b details the magnetic scalar potential, indicating maximum absolute values of 2 A. These simulations elucidate the effect of a continuous magnet on establishing a uniform magnetic field across the entire breadth of the separation channel. Notably, the influence of the neodymium magnet extends beyond the channel directly aligned with the magnet into adjacent channels, albeit with reduced intensity.

Figure 4c,d demonstrate the retention of magnetite nanoparticles through both in silico simulations and experimental tests, specifically within a trial conducted at a flow rate of 2 mL/h and observed at the 10 min mark. In both in silico simulations and experimental tests, the effect of the applied magnetic field manifests in the visible accumulation of magnetite nanoparticles, characterized by their distinct brown hue. This phenomenon is not confined to the channel directly aligned with the magnet but is also observed within the ingress and egress channels. This pattern of nanoparticle retention is related to the magnetic field distribution simulations presented in Figure 4a,b. These panels outlined that the influence of the magnetic field, while the most powerful in the channel parallel to the magnet, indeed permeates the entire channel network, albeit with diminishing intensity. The results from the simulations align with the experimental observations, underscoring the reach of the magnetic field within the device architecture and its role in the effective capture of magnetic nanoparticles.

Figure 4e delineates the performance of the magnetic separation process in terms of recall, precision, and accuracy, integrating data from both in silico simulations and experimental tests. The graph illustrates how the separation efficiency of the device varies with changes in total flow rate (TFR).

At a TFR of 2 mL/h, the device’s capability to segregate magnetic nanoparticles was remarkable, achieving an average recall of 93.3 ± 4%, precision of 95.9 ± 1.2%, and accuracy of 93.4 ± 1.4%. This optimal performance demonstrates the system’s proficiency in nanoparticle retention at lower flow rates. In stark contrast, as the TFR was increased to 20 mL/h, a notable decline in accuracy to 61.3% ± 0.2% was observed in simulations and in the experimental test below 40.1% ± 2.6%. The observed discrepancy between the in silico and experimental results suggests that our computational models might not encapsulate the full spectrum of inter-particle interactions, such as magnetic dipole–dipole attraction, hydrodynamic effects in close-packed conditions, and particle collisions [50,51,52,53]. Additionally, discrepancies can stem from the inherent challenge of accurately simulating the strength and gradient of the magnetic field within the experimental setup [54]. Real-world variations in the magnetic field due to manufacturing tolerances, alignment inaccuracies, and material heterogeneities are complex to model with high fidelity [55]. Consequently, these factors can lead to a divergence in theoretical predictions of particle behavior when compared to their actual trajectories and separation efficiencies observed under experimental conditions.

The trend of diminishing efficiency becomes more acute at the highest TFR of 200 mL/h, where accuracy drops to 35.9% ± 7.1% and recall rates descend to a mere 19.3% ± 8.5% in the experimental test. Such results emphasize the limitations of the magnetic separation device under high-flow conditions, where the disparity between simulated predictions and experimental outcomes widens, illuminating the challenges in modeling and practical application.

This reduction in performance with increasing TFR can be attributed to the dynamic interplay between drag force and magnetophoretic force within the microfluidic device. At lower flow rates, magnetophoretic force—which directs particles toward stronger magnetic fields—predominates, enabling high-precision particle capture. However, as TFR rises, the drag force—propelling particles along the flow path—gains dominance, undermining the magnetophoretic force and reducing particle retention [54,56].

The advancements in the separation capability of the new device configuration are starkly highlighted when compared with previous iterations [57]. Where earlier studies plateaued at recall levels of no more than 50%, the current system substantially exceeds these benchmarks, reaching recall rates as high as 93.3% under the optimal TFR of 2 mL/h. This significant progress, as evidenced by the data, represents a marked improvement in the design and function of the magnetic separation system, as corroborated by the exceptional operational outcomes achieved.

### 3.2. Microparticle Separation

Following the successful assessment of the magnetic separation microfluidic device’s capability at the nanoscale, we extended our investigation to examine its performance in separating larger, micrometer-sized particles. For this phase of the evaluation, we employed a binary separation framework, with magnetite microparticles as the target (positive) population and commercial polystyrene particles (MV-F02, Microvec, Piczów, Poland) labeled with rhodamine B serving as the non-target (negative) fraction.

**Figure 5:**
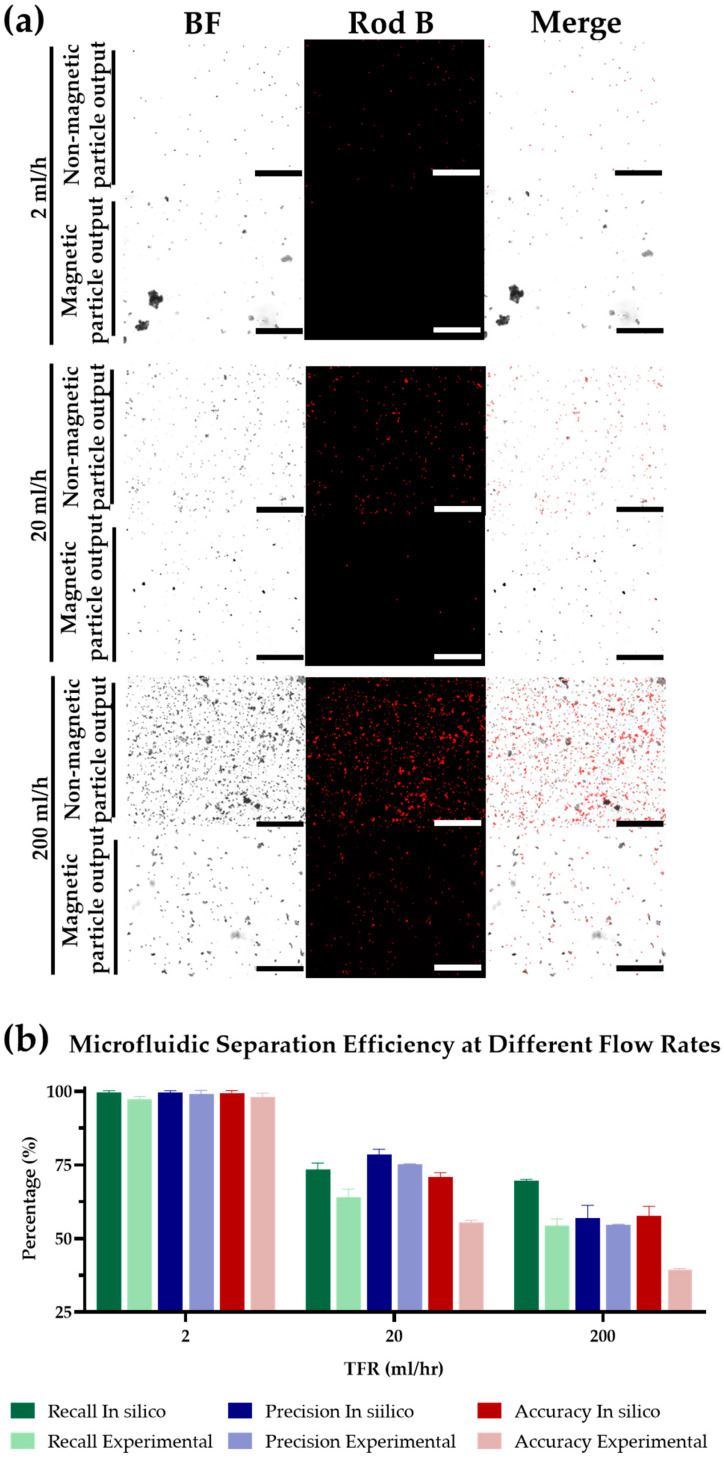
Microparticle separation efficacy: (**a**) Fluorescence microscope images: non-magnetic polystyrene particles exhibit red fluorescence, while magnetite particles are non-fluorescent and visible in the transmitted BF (Bright Field) overlay. Scale-bar 50 μm. (**b**) Analysis of recall, precision, and accuracy, derived from the fluorescence microscope images, illustrating the device’s performance.

The device’s proficiency in microparticle separation was scrutinized at various total flow rates (TFRs) of 2, 20, and 200 mL/h, mirroring the conditions used in the nanoparticle separation tests. This approach allowed for a direct comparison of the device’s performance across different particle size regimes. The corresponding outcomes from the computational and empirical analyses are documented in Figure 5, which shows fluorescence microscopy images where rhodamine B-labeled polystyrene particles emit a distinct red fluorescence, contrasted against the non-fluorescent magnetite particles, which are visible only via transmitted Bright Field (BF) microscopy. These images provide a clear visual representation of the separation achieved by the device, with the two particle types distinctly segregated.

Figure 5b depicts the derived frequency histogram from these microscopy images, quantitatively addressing recall, precision, and accuracy parameters across the different flow rates. Optimal results were observed at a flow rate of 2 mL/h, where the device demonstrated an average recall of 97.3 ± 1%, precision of 99.1 ± 2%, and accuracy of 98.1 ± 1% with minimal deviation between in silico and experimental data. These exceptional performance metrics underscore the device’s high efficiency in separating microparticles under low flow rate conditions. As with the nanoparticle separation study, the device’s effectiveness declined with increased TFR. This trend is attributed to the enhanced drag force at higher flow rates overpowering the magnetophoretic force, a phenomenon consistent across both micro- and nanoparticle regimes.

At a TFR of 20 mL/h, accuracy diminished to 71.0% ± 1% in silico and 55.5% ± 1% experimentally. The most substantial drop occurred at a TFR of 200 mL/h, where accuracy reached 57.7% ± 2% in silico and 39.4% ± 1% experimentally. Interestingly, the comparative analysis of microscale particle separation revealed that the discrepancies between in silico and empirical data were significantly attenuated in comparison to those observed in the nanoparticle separation studies. This phenomenon suggests a more accurate representation of the microscale separation process within the computational models.

One plausible explanation for this improved correlation is the inherent suitability of the Lagrangian approach for modeling the behavior of larger particles [58,59,60]. In microscale simulations, this approach effectively accounts for the individual trajectories of each particle, influenced by the balance of forces acting upon them [61,62]. Due to their larger size, microparticles exhibit more predictable and less erratic motion under the influence of the magnetic field and fluid dynamics, making the Lagrangian models particularly adept at simulating their behavior.

In contrast, nanoparticles, which are influenced by Brownian motion and other stochastic effects, present a greater challenge for computational models, which might not fully encapsulate these complexities [63,64]. Therefore, while the Lagrangian approach provides a good approximation of the microparticle behavior, the nanoparticle dynamics require a more nuanced modeling technique that can accommodate the additional variables influencing their motion.

This assessment underscores the need for specialized modeling techniques for different particle scales to enhance the precision of in silico predictions and their alignment with empirical observations. It also highlights the robustness of our microfluidic device in effectively separating particles across different size regimes, with particularly high efficiency for microparticle separation.

The superior performance in microparticle separation, especially at low flow rates, opens numerous potential applications for this device. These could range from the purification of biological samples, such as isolating specific cell types or removing contaminants from water samples, to industrial applications like the separation of magnetic catalysts or the recovery of magnetic materials from waste streams. The high accuracy and precision demonstrated by the device suggest its potential for use in scenarios where high-purity separation is critical, such as in medical diagnostics or in the preparation of samples for sensitive analytical techniques.

### 3.3. Microparticle Sedimentation in Microchannels

**Figure 6:**
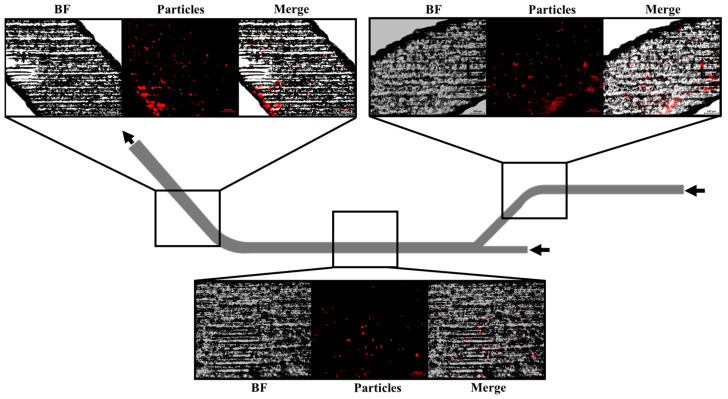
Microparticle sedimentation in microchannels: Bright Field and fluorescent images show minimal sedimentation of rhodamine B-labeled polystyrene microparticles, primarily in curved regions. The sedimentation is minimal and does not significantly obstruct the microchannel or impact the device’s functionality. Black arrows show the direction flow.

The microchannels were thoroughly analyzed and studied after use and washing to identify any microparticles that might have sedimented within them. The microfluidic device was evaluated using a fluorescent microscope, as shown in Figure 6. This figure presents different sections of the microchannel, including Bright Field (BF) images, red fluorescence from the microparticles, which are polystyrene microparticles labeled with rhodamine B, and merged images combining the BF and fluorescent signals.

Figure 6 reveals that some microparticles, identified by their red fluorescence, were indeed sedimented within the microchannel. These sedimented particles are present in a low number, and their distribution is particularly notable in the curved regions of the microchannel. Despite their presence, the overall number of sedimented microparticles is minimal. Therefore, the sedimentation observed does not pose a significant risk of obstructing the microchannel, and it is unlikely to adversely affect the separation mechanism of the microfluidic device. This finding suggests that while some sedimentation occurs, it does not compromise the device’s functionality or efficiency.

### 3.4. Strengths, Weaknesses, Opportunities, and Threats (SWOT)

**Figure 7:**
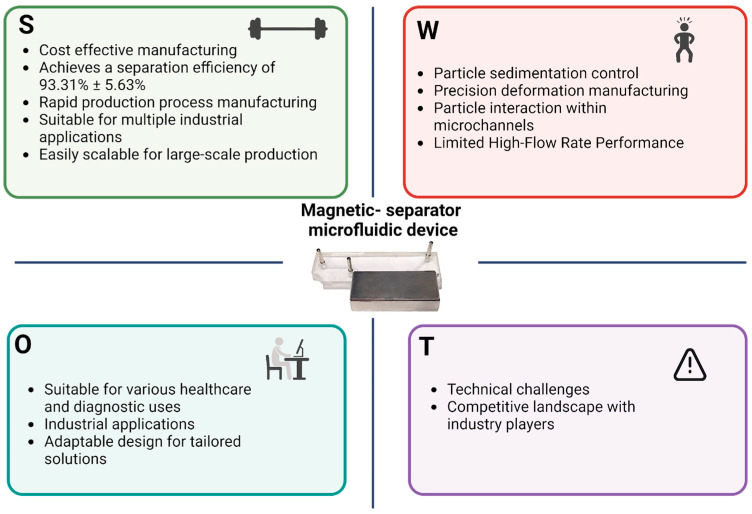
SWOT analysis of the magnetic separator microfluidic device. Strengths (S), weaknesses (W), opportunities (O), and threats (T).

The magnetic separator microfluidic device developed in this article exhibits several strengths, weaknesses, opportunities, and threats, making it a compelling subject for analyzing its potential impact on industrial applications, as presented in the SWOT analysis in Figure 7. One of the key strengths of the device is its high efficiency in separating magnetic nano- and microparticles. The device achieves an impressive accuracy of 95.3% in the separation of magnetic nanoparticles and 92.3% in the purification of magnetic microparticles. This level of performance is a significant improvement over traditional methods, which often struggle to reach such high levels of precision. Another major strength is the low cost associated with the manufacture of the microfluidic device. Priced at less than $1 per unit, the device does not require a cleanroom facility for production, making it both cost-effective and accessible to laboratories worldwide (Table 3). This affordability and ease of manufacturing could democratize the use of advanced microfluidic technologies in diverse research and industrial settings. Additionally, compared to traditional particle purification devices, this microfluidic device accelerates the separation process due to the small channel distances that particles must traverse and the capability to operate in a continuous flow, thereby enhancing efficiency.

However, the device also has notable weaknesses. Its optimal performance is achieved at low flow rates, which limits its ability to process large volumes of magnetic nanoparticles or microparticles quickly. This limitation could hinder its application in scenarios requiring rapid, high-throughput processing. Another weakness is the tendency of particles to sediment in the microchannels at low flow rates, which can affect the device’s usability and consistency over time.

Despite these weaknesses, the microfluidic device offers numerous opportunities. Its low-cost and high-efficiency design make it applicable across a wide range of industries. In biomedical engineering, for example, the device could be used in diagnostic platforms to hyper concentrate patient samples, enhancing the detection of diseases. In materials science, the device could be employed to purify magnetic nanomaterials present in low concentrations, improving the quality and yield of these valuable materials.

However, there are also threats to the widespread adoption of this microfluidic device in industry. One of the primary challenges is the lack of familiarity and acceptance of microfluidic technology within certain industrial sectors. Many industries may be hesitant to adopt microfluidic devices due to a lack of understanding or experience with the technology, which could slow its integration into existing processes.

In summary, the magnetic separator microfluidic device presents a highly promising technology with significant strengths in performance and cost-effectiveness. However, its weaknesses, particularly in handling large volumes at higher flow rates and the potential challenges related to industry acceptance, must be addressed to fully realize its potential in various applications. With continued development and targeted efforts to raise awareness and understanding of microfluidic technologies, this device could play a crucial role in advancing both research and industrial processes.

## 4. Conclusions

The study successfully developed and evaluated a novel microfluidic device for the purification and hyperconcentration of magnetic micro- and nanoparticles. Utilizing a cost-effective laser ablation technique in PMMA, the device eliminates the need for cleanroom facilities, making it more accessible. The device demonstrated robust capabilities in separating magnetic nanoparticles from blue carbon dots and magnetite microparticles from polystyrene microparticles.

For nanoparticle separation, the device achieved up to 93.3% recall and 95.9% precision at an optimal TFR of 2 mL/h, significantly outperforming previous models. This performance vastly exceeds that of our preceding models, which achieved no more than a 50% recall rate [57]. The integration of a continuous magnet is recognized as the pivotal factor enhancing the magnetic separation effect compared to the segmented influence in previous designs. The device’s proficiency at separating micro-sized magnetic particles also peaked at a TFR of 2 mL/h, achieving 98% accuracy in both simulated and experimental conditions. The results highlight the effectiveness of the Lagrangian model in capturing microparticle dynamics, while the behavior of nanoparticles suggests the need for more complex Euler–Euler dispersed phase models for better analysis. This discrepancy between micro- and nanoparticle modeling accuracy provides valuable insights for future improvements in computational simulations of particle behavior in microfluidic devices.

A key finding of this study is the inverse relationship between flow rate and separation efficiency for both micro- and nanoparticles. This relationship is attributed to the balance between magnetophoretic and drag forces, with lower flow rates allowing for more effective magnetic capture. This insight is crucial for optimizing the device’s performance in various applications. The success of this low-cost, high-performance microfluidic device opens up numerous possibilities for its application in various fields. In biomedical research, it could be used for isolating specific cell types or purifying biological samples. In environmental science, it could aid in water purification or the removal of magnetic contaminants. Industrial applications might include the recovery of magnetic catalysts or the separation of magnetic materials from waste streams.

Future work could focus on further optimizing the device design to maintain high separation efficiency at higher flow rates, thereby increasing throughput. Additionally, exploring the device’s performance with a wider range of particle types and sizes could expand its potential applications.

This study underscores the potential of low-cost, non-cleanroom manufacturing techniques to produce high-performance microfluidic devices, thereby expanding their applications in various industrial and research settings.

## Figures and Tables

**Table 1:** List of materials used in the study and their source.

Material	Purity	Supplier	Location
N-[3-Dimethylamino-propyl]-N’-ethyl carbodiimide hydrochloride (EDC)	98%	Sigma-Aldrich	St. Louis, MO, USA
Iron (II) chloride tetrahydrate	98%	Alfa Aesar	Haverhill, MA, USA
Iron (III) chloride hexahydrate	97%	Panreac AppliChem	Barcelona, Spain
N-hydroxysuccinimide (NHS)	98%	Sigma-Aldrich	St. Louis, MO, USA
(3-Aminopropyl) triethoxysilane (APTES)	99%	Sigma-Aldrich	St. Louis, MO, USA
Tetramethylammonium hydroxide pentahydrate (TMAH)	≥97%	Sigma-Aldrich	St. Louis, MO, USA
Rhodamine B	>95%	Sigma-Aldrich	St. Louis, MO, USA
Citric acid	99%	Sigma-Aldrich	St. Louis, MO, USA
Urea	99%	Sigma-Aldrich	St. Louis, MO, USA
Sodium chloride (NaCl)	99.9%	Merck	St. Louis, MO, USA
Polymethyl methacrylate (PMMA) sheets	-	Local distributors	Bogotá, Colombia

**Table 2:** Materials for the manufacturing of the microfluidic device.

Material	Quantity	Cost per Unit/mL (USD)	Total Cost (USD)
Microfluidic connectors	3	USD 0.03	USD 0.09
PMMA Sheets (7.5 cm × 2 cm × 2 mm)	1	-	USD 0.19
PMMA Sheets (7.5 cm × 2 cm × 3 mm)	1	-	USD 0.28
UHU glue	1.5 mL	USD 0.087	USD 0.13
70% *v*/*v* Ethanol	10 mL	USD 0.002	USD 0.02
96% *v*/*v* Ethanol	2 mL	USD 0.015	USD 0.03
**Total cost**			USD 0.74

**Table 3:** Comparative analysis of microfluidic devices in the literature.

Study	Our Work	[65]	[66]	[67]	[68]	[69]	[70]	[71]
**Channel Geometry**		T-shaped	Spiral-shaped, Y-shaped (magnetic section)	Wave	Lineal	Y-shaped	Y-shaped	Y-shaped
**Material**	PMMA	PDMS	PDMS	PDMS	PDMS	PDMS	PDMS	PDMS
**Fabrication technique**	CO_2_ laser	Soft lithography	Soft photolithography	Soft lithography	Soft lithography	Soft lithography	Soft lithography	Soft lithography
**Depth**	1 mm	-	-	20–100 µm	0.1 mm	-	900 µm	100 µm
**Size microfluidic channel**	1 mm	30 µm in thickness	400 µm in width	1–3 mm in width	0.5 mm in width	52 µm in thickness	900 µm in width	<250 µm in width
**Cross section**	Gaussian	Rectangular	Rectangular	Rectangular	Rectangular	Rectangular	Rectangular	Multichannel
**Flow rate**	2–200 mL/h	0.3 µL/min–0.7 µL/min	600–1200 µL/min	-	0.5–2 mL/h	0.5–8 µL/min	3–10 µL/min	-
**Cost per chip**	USD 0.74	>1 USD	>1 USD	>1 USD	>1 USD	>1 USD	>1 USD	>1 USD
**Fabrication time**	30 min	>1 h	2 h 24 min	>1 h	4 h	>1 h	>1 h	>1 h
**Separation efficiency**	93.31% ± 5.63%	88.79–97.36%	93–95%	96.49–98.72%	75–100%	80–86%	97–99%	50–100%
**Particle size**	20 nm–2 µm	0.2–1 µm	7.5–8.7 µm	10 µm	-	5.8 µm and 15.7 µm	2–16 µm	5–18 µm
**Application**	Micro- and nanoparticle purification	Separation and purification of the biological samples of nanometer size	Circulating tumor cells (CTCs) isolation from a blood sample	Magnetophoretic separation of cells	Cell sorting	Cell separation	Cell sorting	Multi-target separation

## Data Availability

The original contributions presented in the study are included in the article/Supplementary Materials; further inquiries can be directed to the corresponding author.

## Supplementary Materials

The following supporting information can be downloaded at: https://www.mdpi.com/article/10.3390/mi15081057/s1, Table S1: Properties of the simulated particles, Figure S1: Hydrodynamic size distribution of the magnetite micro- and nanoparticles.
