# Supplementary material for: Enhancing Magnetic Micro- and Nanoparticle Separation with a Cost-Effective Microfluidic Device Fabricated by Laser Ablation of PMMA
Source: Micromachines (Basel). 2024 Aug 22;15(8):1057. doi: 10.3390/mi15081057 (PMC11356012; doi:10.3390/mi15081057)
Supplement: Supplementary file 1 [file micromachines-15-01057-s001.zip › micromachines-3142981-supplementary.pdf]

micromachines

Brief Report

Enhancing Magnetic Micro- and Nanoparticle Separation with a Cost-Effective Microfluidic Device Fabricated by Laser Ablation of PMMA

Cristian F. Rodríguez [1,2], Paula Guzmán-Sastoque [1], Carolina Muñoz-Camargo [1], Luis H. Reyes [3], Johann F. Osma [1,4] and Juan C. Cruz [1,3,*]

1 Department of Biomedical Engineering, Universidad de los Andes, Cra. 1E No. 19a-40, Bogotá 111711, Colombia; cf.rodriguez@uniandes.edu.co (C.F.R.); pa.guzmans@uniandes.edu.co (P.G.-S.); c.munoz2016@uniandes.edu.co (C.M.-C.); jf.osma43@uniandes.edu.co (J.F.O.)
2 Neuroscience Group of Antioquia, Cellular and Molecular Neurobiology Area, School of Medicine, University of Antioquia, Medellin 050010, Colombia
3 Grupo de Diseño de Productos y Procesos (GDPP), Department of Chemical Engineering, Universidad de los Andes, Cra. 1E No. 19a-40, Bogotá 111711, Colombia; lh.reyes@uniandes.edu.co
4 Department of Electrical and Electronic Engineering, Universidad de los Andes, Cra. 1E No. 19a-40, Bogotá 111711, Colombia
* Correspondence: jc.cruz@uniandes.edu.co

Supplementary material

Table S1 Properties of the simulated particles. References of the parameters used in literature [1,2]

Simulation Scenario	Particle Type	Density (kg/m³)	Relative permeability	Diameter (Mean , Deviation)	Results
Magnetic separation nano particle	Nano magnetite	5180	2.5	90 nm, 10 nm	Figure 2
	Blue carbon dots	1500	-	3 nm, 1nm	
Magnetic separation Micro particle	Polystyrene Microparticles	1500	-	2 μm, 1 μm	Figure 3
	Micro magnetite	5180	2.5	3 μm, 1 μm	

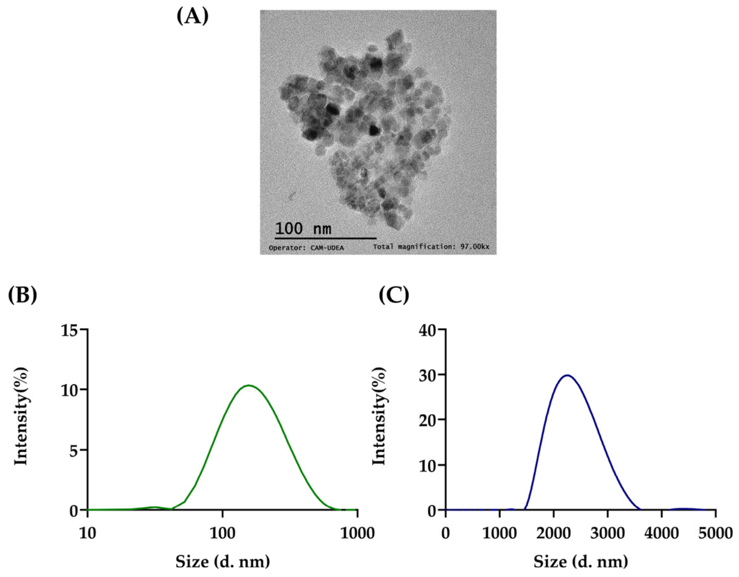

Figure S1. (A) TEM image of the magnetite nanoparticles. Scale bar 100 nm. (B) Hydrodynamic size distribution of magnetite nanoparticles. (C) Hydrodynamic size distribution of magnetite microparticles.

References

(1) Torres, C. E.; Cifuentes, J.; Gómez, S. C.; Quezada, V.; Giraldo, K. A.; Puentes, P. R.; Rueda-Gensini, L.; Serna, J. A.; Muñoz-Camargo, C.; Reyes, L. H.; Osma, J. F.; Cruz, J. C. Microfluidic Synthesis and Purification of Magnetoliposomes for Potential Applications in the Gastrointestinal Delivery of Difficult-to-Transport Drugs. *Pharmaceutics* **2022**, *14* (2), 315. https://doi.org/10.3390/pharmaceutics14020315.

(2) Rodríguez, C. F.; Guzmán-Sastoque, P.; Gantiva-Diaz, M.; Gómez, S. C.; Quezada, V.; Muñoz-Camargo, C.; Osma, J. F.; Reyes, L. H.; Cruz, J. C. Low-Cost Inertial Microfluidic Device for Microparticle Separation: A Laser-Ablated PMMA Lab-on-a-Chip Approach without a Cleanroom. *HardwareX* **2023**, *16*, e00493. https://doi.org/10.1016/j.ohx.2023.e00493.
